# The first Dutch *SDHB *founder deletion in paraganglioma – pheochromocytoma patients

**DOI:** 10.1186/1471-2350-10-34

**Published:** 2009-04-15

**Authors:** Jean-Pierre Bayley, Anneliese EM Grimbergen, Patrick A van Bunderen, Michiel van der Wielen, Henricus P Kunst, Jacques W Lenders, Jeroen C Jansen, Robin PF Dullaart, Peter Devilee, Eleonora P Corssmit, Annette H Vriends, Monique Losekoot, Marjan M Weiss

**Affiliations:** 1 Department of Human Genetics, Leiden University Medical Center, Leiden, The Netherlands; 2 Department of Clinical Genetics, Leiden University Medical Center, Leiden, The Netherlands; 3 Department of Otorhinolaryngology, Leiden University Medical Center, Leiden, The Netherlands; 4 Department of Endocrinology, Leiden University Medical Center, Leiden, The Netherlands; 5 Department of Internal Medicine, Radboud University Nijmegen Medical Center, Nijmegen, The Netherlands; 6 Department of Endocrinology, University of Groningen and University Medical Center Groningen, Groningen, The Netherlands

## Abstract

**Background:**

Germline mutations of the tumor suppressor genes *SDHB, SDHC *and *SDHD *play a major role in hereditary paraganglioma and pheochromocytoma. These three genes encode subunits of succinate dehydrogenase (SDH), the mitochondrial tricarboxylic acid cycle enzyme and complex II component of the electron transport chain. The majority of variants of the SDH genes are missense and nonsense mutations. To date few large deletions of the SDH genes have been described.

**Methods:**

We carried out gene deletion scanning using MLPA in 126 patients negative for point mutations in the SDH genes. We then proceeded to the molecular characterization of deletions, mapping breakpoints in each patient and used haplotype analysis to determine whether the deletions are due to a mutation hotspot or if a common haplotype indicated a single founder mutation.

**Results:**

A novel deletion of exon 3 of the *SDHB *gene was identified in nine apparently unrelated Dutch patients. An identical 7905 bp deletion, c.201-4429_287-933del, was found in all patients, resulting in a frameshift and a predicted truncated protein, p.Cys68HisfsX21. Haplotype analysis demonstrated a common haplotype at the *SDHB *locus. Index patients presented with pheochromocytoma, extra-adrenal PGL and HN-PGL. A lack of family history was seen in seven of the nine cases.

**Conclusion:**

The identical exon 3 deletions and common haplotype in nine patients indicates that this mutation is the first Dutch *SDHB *founder mutation. The predominantly non-familial presentation of these patients strongly suggests reduced penetrance. In this small series HN-PGL occurs as frequently as pheochromocytoma and extra-adrenal PGL.

## Background

Paragangliomas occur as tumors of parasympathetically innervated head and neck paraganglia (HN-PGL), as intra-abdominal and thoracic extra-adrenal paragangliomas of the sympathetic paraganglia, and as pheochromocytomas (PCC) of the adrenal medulla. Sympathetic paragangliomas may present clinically with hypertension, sweating and palpitations due to catecholamine excess, and especially in cases with extra-adrenal localization, they may be metastatic and aggressive. HN-PGL usually follows a mild course but may lead to significant morbidity due to compromised function of cranial nerves.

Although many paragangliomas are apparently sporadic (i.e. no known family history), many patients will carry a germline mutation, and worldwide up to 30% of all cases can be shown to have familial antecedents [1].

The identification in HN-PGL families of germline mutations of *SDHD *(succinate dehydrogenase, subunit D) [2] was soon followed by the identification of germline mutations in *SDHB *[3] and *SDHC *[4]. These three genes encode subunits of the mitochondrial tricarboxylic acid cycle enzyme, succinate dehydrogenase (SDH). SDH also acts as the complex II component of the electron transport chain, locating SDH at the center of cellular metabolism.

Despite the fact that SDH is thought to act solely as a unified protein complex, mutations of subunit genes lead to striking differences in clinical phenotype. While mutations of *SDHD *are associated predominantly with HN-PGL, frequently multifocal and generally non-metastatic,*SDHB *mutation carriers frequently present with PCCs and extra-adrenal paragangliomas, and mutations of *SDHB *are more often found in patients with aggressive, metastatic disease [5]. Until recently mutations of *SDHC *were exclusively associated with HN-PGL, but have now also been identified in patients with PCC [6,7].

The majority of mutations of the SDH genes described in the SDH mutation database [8] are missense and nonsense mutations (n = 225). To date only ten distinct large deletions of the SDH genes have been described.

Although nearly all familial paraganglioma in the Netherlands is accounted for by the Dutch *SDHD *founder mutations p.Asp92Tyr and p.Leu139Pro [9], several Dutch families carrying an *SDHB *mutation were recently identified [10]. Here we describe the results of *SDHB *gene deletion scanning of 126 paraganglioma-PCC patients. Nine apparently unrelated Dutch patients all showed deletions of exon 3 of the *SDHB *gene. In order to determine if exon 3 is affected by a deletion hotspot, we proceeded to the molecular characterization of the deletion, mapping the breakpoint in each patient, and used haplotype analysis to determine whether the patients share any common haplotype, which would suggest a single novel founder mutation. In addition, the clinical phenotype of these patients is described.

## Methods

### Patients

Between 2000 and 2008, a total of 251 index patients with either a paraganglioma or PCC were referred for molecular testing of the *SDHD*/*B/C *genes to the Molecular Genetics Laboratory at the Leiden University Medical Center, The Netherlands. Informed consent was obtained for DNA testing according to protocols approved by LUMC Ethics Review Board. DNA was available from 126 index patients who tested negative for *SDHD *point mutations and in whom point mutations of *SDHB *and *SDHC *were, in most cases, also excluded.

### Multiplex ligation dependent probe amplification

MLPA was carried out with the P226 MLPA kit , containing probes for all exons of the *SDHB*, *SDHC *and *SDHD *genes, as well as probes located in the promoter of each gene (27 different probes). MLPA analysis was performed according to the MRC Holland protocol [11] except that all reagents in the kit were used at 1/2 of the recommended volume and hybridization time was reduced from 16 to 2.5 hours. No difference in results was seen compared to recommended conditions.

### Haplotype analysis

Analysis of haplotypes by polymorphic di- and tetra nucleotide markers (microsatellite markers) was performed according to standard procedures (details available upon request), using the following markers: D1S436, D1S2697, D1S170, D1S3669, D1S2826 and D1S2644. The distance between the last and the first marker is ~3.35 Mb. In addition, intragenic SNPs were sequenced to refine the haplotype (Table 1). The frequency of marker alleles in the Dutch population was determined in 24 healthy controls.

**Table 1:** Name and physical location of chromosome 1 microsatellite markers and intragenic SNPs used for haplotyping

Marker name (in chromosomal order)	Chromosomal location (UCSC Genome Browser. Human Mar. 2006 Assembly)
D1S436	15643044

D1S2697	16192037

D1S170	17070023

***SDHB *gene**	**17217812**

rs12045097	17218463

rs2871775	17218491

rs978528	17222384

rs2235931	17222541

rs2235930	17222729

rs2647162	17222829

rs12142244	17223517

rs2235929	17223682

rs7550829	17230163

rs2746467	17230201

rs11577071	17231760

rs4920390	17231880

rs10887990	17231972

rs11203284	17242538

rs11203285	17242706

rs10887992	17242799

rs10887993	17242827

c.200+987C>T	

rs11203287	17244983

rs9435747	17245053

rs11582579	17245097

rs6690934	17245208

rs7536679	17247249

rs7545499	17247274

rs7545518	17247328

***SDHB *gene**	**17253252**

D1S3669	17556185

D1S2826	18205820

D1S2644	18799054

### Breakpoint characterization

Long range PCR using the primers F2 (5'-TCT GTT GTG CCA GCA AAA TG-3') and R4 (5'-CAA ATC CTG CCC TGA AAA AC-3') was carried out using the Takara LA Taq kit (Takara Bio Inc., Lucron Bioproducts B.V., Gennep, The Netherlands) following the manufacturers recommendations. The resulting PCR fragment of 8.5 kb in the patients carrying the deletion was subjected to restriction mapping using the following twelve enzymes: *Alw*44I, *Bgl*I, *Bgl*II, *Bsa*HI, *Bsp*EI, *Eco*RI, *Mph*1103I, *Nde*I, *Sac*I, *Sca*I, *Sma*I, *Xba*I. Analysis of the resulting restriction patterns narrowed the specific region of the deletion, and was followed by the design of primers 2162 (5'-CCA GTC CAT GAA AGG CAA-3') and 2164 (5'-GCT CCA TGT GTC ACG TGT TT-3'). This allowed the amplification of a 1.6 kb fragment in patients carrying the deletion but not in healthy controls. The PCR product was sequenced, and analyzed using the Multalin program . Sequence analysis of the *SDHB *gene was performed according standard procedures (details available upon request), using the NT_004610.18 reference sequence.

## Results

### MLPA analysis

MLPA allows the detection of large deletions, and is based on the quantification of multiplexed amplified DNA fragments. It was first described by Schouten *et al*. in 2002 [11] and due to reliability and ease of use has been widely adopted in both research and diagnostic settings.

**Figure 1:**
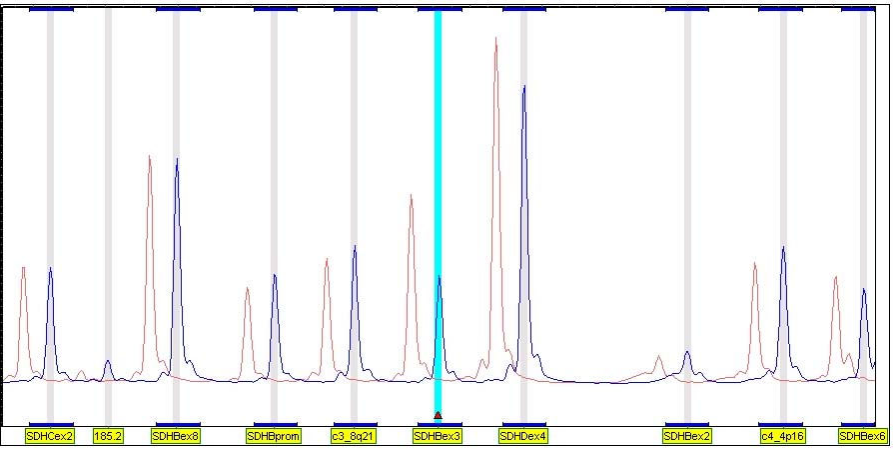
**An example of MLPA analysis of the *SDHB*, *SDHC *and *SDHD *genes**. Only some exons of each gene can be seen in this view but all are included in the kit (P226 MLPA kit), plus the promoters of each gene, and several control fragments located on various chromosomes. Red peaks show the average of normal control DNA and blue peaks represent the DNA of the patient. The deletion of exon 3 of *SDHB *can clearly be seen (small red diamond).

**Figure 2:**
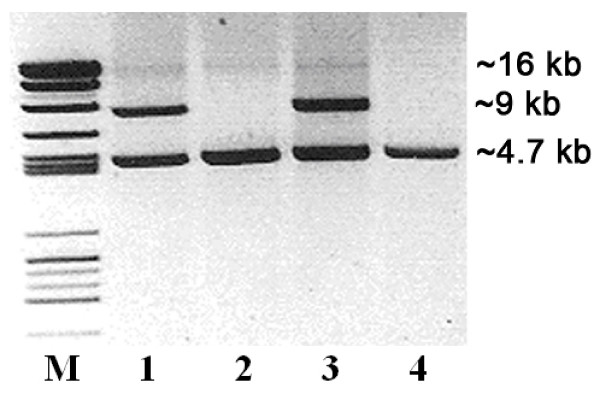
**Long range PCR of the exon 3 region of *SDHB***. Lanes 1 & 3. Patients carrying deletions showed an anomalous fragment of ~9 kb. Lanes 2 & 4. Two healthy controls lack the anomalous ~9 kb fragment. The normal fragment (~16 kb) is weakly visible in all lanes. A PCR control fragment of ~4.7 kb is also visible in all lanes.

All 251 index patients were screened for germline mutations in *SDH *genes by sequence analysis. A pathogenic mutation was identified in 125 patients (50%). MLPA analysis was performed in 126 mutation negative patients, and included all exons and the promoter of the *SDHB*, *SDHC *and *SDHD *genes. Deletions of the *SDHB *gene were detected in nine patients, all affecting exon 3 (figure 1). Four deletions were identified in the *SDHC *and *SDHD *genes and will be described elsewhere. In the entire series 13 of 251 patients (5%) were found to have deletions, representing approximately 10% of all mutations found. To further characterize the *SDHB *deletions and confirm the MLPA results, a long range PCR flanking exon 3 was designed. While the expected normal fragment (~16 kb) could barely be detected, all patients carrying exon 3 deletions showed a shorter fragment of ~9 kb (figure 2).

### Breakpoint characterization

Restriction mapping of the 9 kb *SDHB *fragment was carried out using twelve enzymes. Analysis of restriction patterns narrowed the breakpoints to small regions of intron 2, approximately 4.5 kb upstream of exon 3 and to a region 3.5 kb downstream, in intron 3. Primers were designed around the expected site of the deletion, and a 1.6 kb fragment could be amplified in all patients carrying the deletion, indicating that all deletions were either identical or mapped to a small and specific region, which would suggest a mutation hotspot.

**Figure 3:**
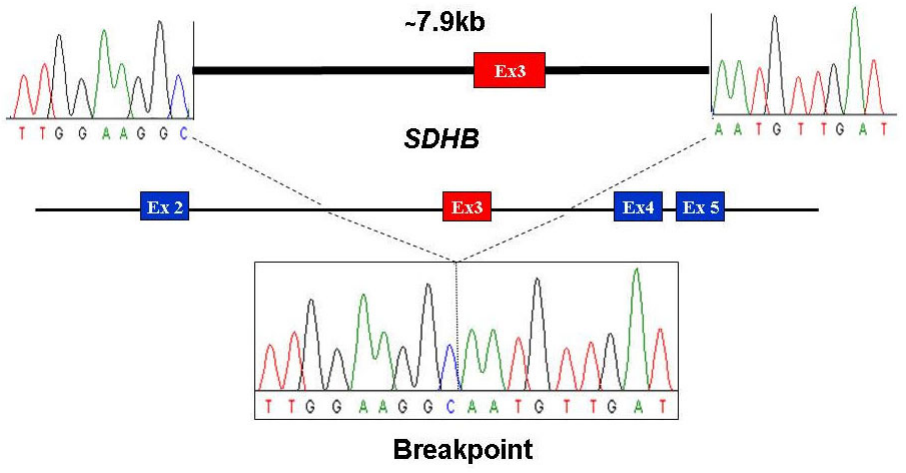
**Characterization of the exon 3 *SDHB *breakpoint**. The lower box shows a patient sequence spanning the deletion. The upper figure indicates the approximate location of each portion of the sequence in respective introns in the normal undeleted gene sequence.

Sequencing of the 1.6 kb PCR product revealed identical breakpoints in all samples, resulting in a deletion of 7905 bp, including exon 3 (figure 3), suggesting a single founder mutation, identical by descent. Following HGVS cDNA nomenclature, the deletion is correctly described as c.201-4429_287-933del. The deletion of exon 3 is predicted to result in a frameshift at the DNA level and a truncated protein, p.Cys68HisfsX21.

Although analysis of the surrounding sequence revealed that the upstream breakpoint is located in an AluSz repeat, the downstream breakpoint is located in unique sequence, rather than in an Alu repeat. Thus there are no repeat sequences or sequence similarity around the breakpoint that would suggest a mechanism of deletion or location of a mutational hotspot.

### Haplotype analysis

Although the patients had no apparent family connection, all are natives of the Netherlands and share an identical *SDHB *deletion.

**Figure 4:**
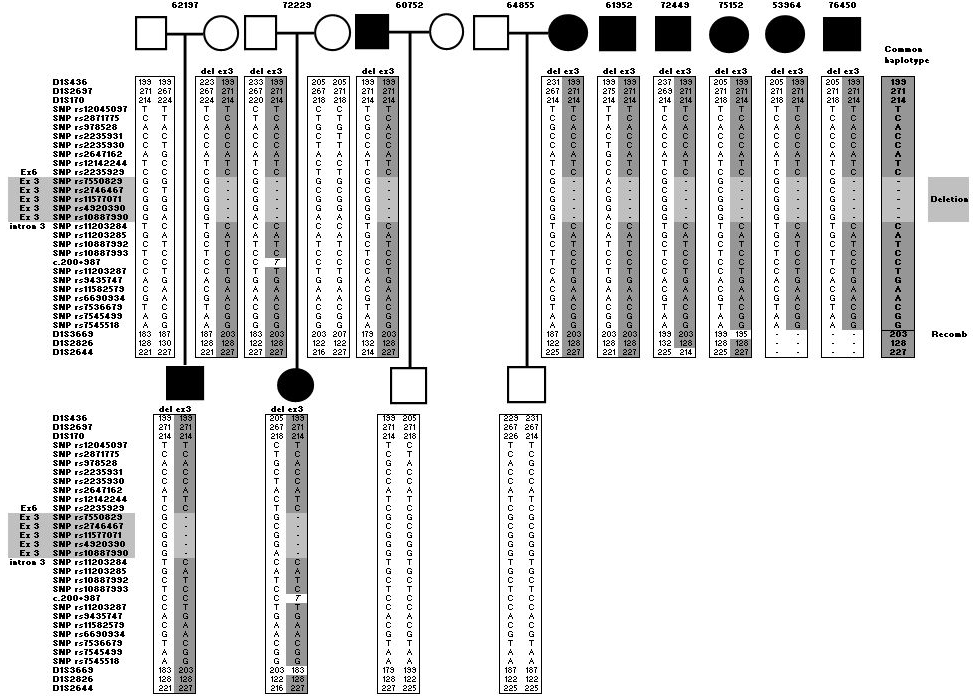
**Haplotype analysis of patients carrying the *SDHB *exon 3 deletion**. Microsatellites flanking *SDHB *and intragenic SNPs demonstrate a common haplotype. The common haplotype and the region deleted are indicated.

Therefore we carried out haplotype analysis with six di- and tetra nucleotide polymorphic markers surrounding the *SDHB *gene. Haplotyping was refined by typing additional intragenic SNPs. A common haplotype could be deduced in all patients (figure 4), indicating descent from a common ancestor. The marker haplotype formed by D1S436, D1S2697, and D1S170 has a frequency of 1% in the Dutch population, and the likelihood of nine unrelated cases carrying this haplotype is ~1.4 × 10^-18^. Certain haplotypes have apparently mutated or recombined in selected patients, an indication that they are only distantly related.

### Clinical data

Patient BD1 presented with a hard mass in the neck at 41 years of age, initially suspected to be an aneurysm, but no other symptoms. MRI imaging revealed a large and highly vascular mass, 4 × 2 cm, at the carotid bifurcation, partially displacing the larynx. A second tumor was visible above the primary mass, apparently a secondary process of the carotid body tumor. Biopsy and pathological evaluation confirmed the diagnosis.

Patient BD2 was diagnosed with both a malignant extra-adrenal paraganglioma and a parathyroid tumor at the age of 60. The patient's brother was diagnosed at the age of 50 with a PCC, and their mother had possibly been treated for a malignant PCC.

Patient BD3 presented at the age of 35 with a right sided jugulotympanic paraganglioma, in combination with raised dopamine levels. MRI of the head and neck revealed no further tumors. A partial resection was carried out in 2007, following complaints of reduced hearing and tinnitus. MRI of the head and neck revealed no further tumors. Although increased urinary dopamine excretion indicated a possible occult sympathetic paraganglioma, MRI of the thorax and abdomen revealed no further tumors. The patient had a negative family history.

Patient BD4 presented with a retroperitoneal paraganglioma directly under the diaphragm and a paraganglioma of the urinary bladder at the age of 28. No other tumors were found. A recent MRI scan, nine years after the initial treatment, revealed no further tumors. The patient had a clinically negative family history, and although the patient's mother also has the mutation, she remains unaffected at the age of 66.

A female patient BD5, presented with bilateral carotid body paraganglioma at 51 yrs, and 15 years later the patient was found to have raised urine normetanephrine and dopamine levels. In addition to visualization of the largest left-sided carotid body paraganglioma by ^111^In-octreotide scintigraphy, DOPA-PET and FDG PET-scanning revealed a suspicious lesion in the abdomen, suggesting co-existing extra-adrenal paraganglioma. Due to the risk of co-morbidity, the lesions are being followed conservatively.

Patient BD6 presented with a malignant extra-adrenal paraganglioma with bone metastases at the age of 12, and a pituitary tumor. The paternal grandfather is known to have had a pituitary tumor. The family history is negative for paraganglioma.

Patient BD7, a 55-year old male, presented with a left sided carotid body paraganglioma, which was resected at the age of 50. To date there is no increased excretion of urinary catecholamine's or their O-methylated metabolites. A recent MRI of the thorax and abdomen revealed no additional paragangliomas. No family history was available.

Patient BD8 presented with a jugulotympanic paraganglioma at the age of 18. Recently, at the age of 51 years, MRI of the head and neck and CT of the thorax revealed that the patient had developed no further tumors. No clear family history was available.

Patient BD9 was recently diagnosed, at the age of 28, with a retroperitoneal presacral paraganglioma without raised catecholamine levels. MIBG, DOPA-PET and FDG of the head, neck and thorax revealed no further tumors. The family history is negative.

Clinical details for all nine patients are shown in overview (Table 2).

**Table 2:** Clinical summary of patients and family members with the *SDHB *exon 3 deletion

**Patient ID**	**Age at Diagnosis**	**Gender**	**Family Clinical History**	**Clinical Features**
BD1	41	Female	Negative	Carotid body paraganglioma, left

BD2	NA	Female	Mother & brother with pheochromocytoma	Extra adrenal pheochromocytoma Parathyroid tumor

BD3	35	Male	Negative	Jugulotympanic paraganglioma with raised catecholamine levels. No evidence of pheochromocytoma

BD4	28	Male	Negative	Retroperitoneal paraganglioma and paraganglioma of the urinary bladder

BD5	51	Female	NA	Bilateral carotid body paraganglioma. Raised urine normetanephrine and dopamine levels, possible extra-adrenal paraganglioma

BD6	12	Female	Negative	Malignant extra-adrenal paraganglioma with bone metastases. Pituitary tumor. Paternal grandfather had pituitary tumor.

BD7	50	Male	Negative	Carotid body paraganglioma, left

BD8	18	Female	NA	Jugulotympanic paraganglioma

BD9	28	Male	NA	Retroperitoneal paraganglioma

## Discussion

While the majority of SDH-related hereditary HN-PGL and PCC patients carry missense and nonsense mutations, a significant proportion of patients may carry whole gene or exon deletions. In the entire current series of patients 13 of 251 (5%) were found to have deletions, representing 10% of all mutations found. These data have clear implications for diagnostic DNA screening and indicate that deletion scanning such as MLPA should be considered once patients have tested negative by sequencing.

Ten deletions affecting SDH genes have been described to date. McWhinney *et al*. first described deletions of *SDHB *and *SDHD *in two families in 2004 [12]. While a large *SDHD *deletion was associated with HN-PGL in one family, a deletion of exon 1 of *SDHB *in a Brazilian family resulted in extra-adrenal PGL, together with HN-PGL. Baysel *et al*. described a family carrying a deletion affecting exon 6 of *SDHC*, and including five affected members, all with HN-PGL [13]. Cascon *et al*. subsequently described deletions of *SDHB *in families from the Iberian peninsula, [14] identifying the first whole gene deletion of *SDHB *and a deletion of exon 1. This latter deletion was shown to be a founder on further characterization and an additional unique exon 1 deletion was identified in a French family [15]. These patients showed predominantly retroperitoneal PGL, but two cases from the French family developed adrenal PCC.

Amar *et al*. have recently described a novel deletion of *SDHB *in a patient studied in relation to metastatic paraganglioma [16]. This deletion was not further characterized. Fish *et al*. reported a mixed picture of both HN-PGL and extra-adrenal PGL in a family with a deletion of exon 3 of *SDHD *[17]. Pasini *et al*. have described a patient who presented at 9 years of age with multiple GISTs, and by the age 16 had developed multiple retroperitoneal paragangliomas. This patient showed a large deletion affecting *SDHB*, but as no tumor material was available, the relation of *SDHB *to the GIST could not be demonstrated [18]. Pigny *et al*. have reported a deletion of *SDHB *exons 7 & 8 in a patient with bilateral pheochromocytoma [19].

The clinical features of the *SDHB *exon 3 deletion patients described in this report differ from most of the reports described above in the higher than expected frequency of HN-PGL. Previous studies have shown that *SDHB *mutations predominantly predispose to abdominal or thoracic paragangliomas and adrenal PCC [20], but as Leiden is a national referral centre for HN-PGL, a referral bias may be operating.

Although relatively few deletions have thus far been described, the current picture does not indicate that the phenotype caused by deletions differs substantially from that of missense and nonsense mutations. While mutations of *SDHB *and *SDHD *show an intriguing divergence in related phenotypes despite the intimate association of the protein subunits, gene deletions are unlikely to show specific genotype-phenotype effects, relative to truncating or missense mutations, because all mutations are assumed to result in loss of protein function.

The best current estimates of penetrance for *SDHB *mutations are 77% at 50 years [20] and approximately 60% at 50 yrs [5]. These figures are based largely on index cases, as few patients present in the context of extended families. Such estimates are known to exaggerate penetrance, and more accurate estimates will require large SDHB-related families, and the inclusion and detailed clinical screening of apparently unaffected mutation carriers in addition to patients.

The only large family described to date [21] suggests a much lower penetrance but there was no detailed clinical screening of mutation carriers and the authors made no attempt to estimate penetrance. The fact that *SDHB *germline mutation carriers often present as apparently non-familial cases has not escaped various authors [5,22-24] and even proven founder mutations of *SDHB *may initially present as isolated families [15] (and this study).

In contrast, *SDHD*-related mutations show a very high penetrance, over 80% at 50 years [5], and also show a striking and unique imprinted or parent-of-origin inheritance [25], showing almost complete penetrance with paternal inheritance, while mutation carriers via the maternal line remain tumor-free throughout life. This is in sharp contrast with the *SDHB *and *SDHC *genes, located on chromosome 1, which do not show parent-of-origin inheritance. All evidence indicates that it is not the *SDHD *gene itself which is imprinted [2,26] but that some additional locus on chromosome 11 is involved.

The major imprinted locus of the human genome is on chromosome 11p15.5, and we have proposed a model in which an imprinted and maternally expressed gene on chromosome 11p15.5 must be lost together with the normal maternal *SDHD *allele (the paternal allele is inactivated by a germline mutation) prior to initiation of tumorigenesis [26]. This mechanism, recently referred to as the 'Hensen model' [27], is supported by additional data for chromosome 11 [28] and may also play a role in chromosome 3-linked *VHL *PCC [29,30].

This model could provide a genetic explanation for the reduced penetrance of *SDHB *mutations, in which both *SDHB *and the modifier must be lost, requiring loss on two separate chromosomes; intrinsically less likely than a single genetic event (whole chromosome loss) which has been shown to be the mechanism in SDHD tumors [26,28]. Alternatively or additionally, the difference in penetrance may have a (partly) biochemical explanation, related to the difference in function of the SDHD and SDHB subunits, the former principally structural, the latter catalytic. A unique biochemical effect of *SDHB *mutations seems likely in the light of the differing location and often aggressive behavior of tumors, and the high number of clinically penetrant mutations identified. No current model of PGL tumorigenesis can explain these phenomena [31,32].

## Conclusion

We describe the first Dutch founder mutation of *SDHB*, a novel deletion of exon 3. Index patients presented with PCC, extra-adrenal PGL as well as HN-PGL. Lack of a clear family history in seven out of nine cases strongly indicates reduced penetrance. Family studies will be extended to further delineate penetrance and expression.

## Competing interests

The authors declare that they have no competing interests.

## Authors' contributions

JPB collected and analyzed the data, co-designed the study, and wrote the manuscript. MMW and ML edited and analyzed the data, co-designed the study, and co-wrote the manuscript. AHV, EPC, and PD designed and implemented the study, and contributed to the manuscript. AEMG, PB, MW, HPK, JJ, JWL, RPFD and GPWG collected and analyzed the data. All authors read and approved the final manuscript.

## Pre-publication history

The pre-publication history for this paper can be accessed here:


